# Age-related cerebral changes during different n-back tasks: a functional near-infrared spectroscopy study

**DOI:** 10.3389/fnagi.2024.1437587

**Published:** 2024-10-16

**Authors:** Shizhe Zhu, Qinglei Wang, Chaojie Kan, Ayan Geng, Youxin Sui, Ren Zhuang, Yi Zhu, Tong Wang, Lan Zhu, Chuan Guo

**Affiliations:** ^1^Department of Rehabilitation Medicine, The First Affiliated Hospital of Nanjing Medical University, Nanjing, China; ^2^Department of Rehabilitation Medicine, Changzhou Dean Hospital, Changzhou, China; ^3^Department of Rehabilitation Sciences, The Hong Kong Polytechnic University, Kowloon, Hong Kong SAR, China

**Keywords:** fNIRS, aging, n-back, cerebral activation, working memory

## Abstract

**Background:**

The n-back task is a widely used paradigm to assess working memory and is commonly applied in research on age-related cognitive decline. However, studies utilizing functional near-infrared spectroscopy (fNIRS) to explore this area are limited.

**Objective:**

This study aims to investigate age-related differences in brain activation during the n-back task using fNIRS.

**Methods:**

fNIRS data were collected from 18 elderly and 19 young participants while performing different n-back tasks. Brain activation patterns and peripheral performance were compared between the two groups.

**Results:**

Significant differences in brain activation patterns were observed between elderly and young participants. Under the 3-back condition, the older group exhibited reduced activation in brain regions adjacent to prefrontal cognitive areas compared to the younger group. Additionally, the older group’s performance plateaued at the 2-back level, along with a decline in prefrontal activation.

**Conclusion:**

These findings may suggest potential markers for cognitive decline, providing a new target for future screening.

## 1 Introduction

The global trend of population aging is becoming increasingly severe, with age-related cognitive decline issues being particularly prominent (Krivanek et al., 2021). Even in the absence of pathological conditions (Harada et al., 2013), elderly individuals experience a certain degree of decline in functions such as memory (Kronovsek et al., 2021), execution (Causse et al., 2019), visual–spatial function (Haberstumpf et al., 2020). This decline may be closely related to anatomical and functional changes in the brain (Grady, 2012), highlighting the importance of exploring the neural mechanisms behind cognitive decline.

Previous studies using positron emission tomography and functional magnetic resonance imaging (fMRI) have identified distinct brain activity patterns correlating with cognitive decline between healthy young and elderly individuals (McDonough et al., 2022). Specifically, older adults show a decrease in brain activity specificity (Reuter-Lorenz and Park, 2014; Koen and Rugg, 2019; Ota and Shah, 2022). This decline in neural distinctiveness, referred to as dedifferentiation, which is marked by either increased activation or attenuation of brain regions (Park et al., 2012), has frequently been linked to poorer cognitive performance (Li et al., 2001; Park et al., 2012; Zhao et al., 2021).

The n-back task, a cognitive assessment frequently used in neuroscience and psychology research, evaluates working memory and cognitive control (Bopp and Verhaeghen, 2020). In this task, participants are presented with a series of stimuli, such as letters, numbers, or shapes, one at a time. Their goal is to indicate whether the current stimulus matches one presented “n” steps back in the sequence. Our trial uses the numeric n-back paradigm, where participants indicate if the current stimulus matches one presented “n” steps back in the sequence, for example, in a 2-back task, participants must remember and compare each stimulus with the one presented two steps earlier. They may be asked to respond by pressing a button or indicating “yes” if there is a match. Increasing “n” increases the task difficulty and number of processing steps. The n-back task demands both passive maintenance and executive processing operations, particularly under higher task loads. Hence, it proves highly appropriate for cognitive aging research (Vermeij et al., 2012).

In fMRI studies utilizing the n-back paradigm, aberrant activation patterns in the prefrontal cortex (PFC) are correlated with cognitive decline in elderly individuals (Salthouse, 1996; Owen et al., 2005). Although fMRI is relatively mature in n-back paradigm research, its application in n-back tasks still presents certain limitations. First, the machine itself is costly, along with high operational expenses. Second, the testing process is inconvenient, especially for people with claustrophobia or metallic implants. Moreover, noise from the fMRI machine could interfere with the activation of the left dorsolateral PFC (DLPFC) (Rovetti et al., 2021). Therefore, functional near-infrared spectroscopy (fNIRS) appears to be a valuable tool due to its affordability, speed, convenience, and ability to monitor brain activity in natural settings. Many studies have verified the application of fNIRS in cognitive aging research, reporting an age-related decline in prefrontal activity during tasks involving associative memory (Talamonti et al., 2020), Stroop task (Takeda et al., 2017), postural control (de Rond et al., 2023), or dual-task walking (Nóbrega-Sousa et al., 2020). Moreover, an age-related decline in prefrontal activity was reported during a verbal-fluency task (Herrmann et al., 2006), with young adults showing left hemispheric lateralization, which was not reported in older adults; older adults additionally recruited the left inferior frontal cortex to compensate for age-related decline. Currently, there are few studies that focus on differences of prefrontal activation between older and younger adults when performing n-back tasks of varying difficulty. Currently, there is considerable variability in experimental designs and research tools across different studies. First, the task design differs. For instance, Vermeij et al.’s (2012) study observed three tasks: 0-, 1-, and 2-back. The 0-back task primarily involves judgment without engaging working memory and may not reveal significant age-related differences between healthy young and older adults, whereas the 2-back and 3-back tasks require higher levels of working memory. In these tasks, participants are required to remember previous stimuli in a series and compare them, demanding more cognitive resources and attention, thus likely to be more susceptible to age-related differences. Second, the arrangement of fNIRS probes can vary significantly across studies, making it difficult to compare findings (Ranchod et al., 2023). Therefore, further research is needed to address these inconsistencies.

The present study aimed to investigate how prefrontal brain activity changes with increasing working memory load in both young and older adults. Based on theories of prefrontal compensatory mechanisms in aging and similar fMRI studies (Scheller et al., 2017; Suzuki et al., 2018), we hypothesize that, compared with young adults, older adults will show increased prefrontal recruitment during easier tasks but decreased activation during more difficult tasks. To test this hypothesis, we measured prefrontal activation during a visual n-back task using fNIRS.

## 2 Methods

### 2.1 Participants

The inclusive criteria were as follows: (1) age 20–35 years for the younger group (YG) and 60–75 years for the older group (OG; most participants aged >75 years recruited in the pilot trial were unable to understand these tasks); (2) Montreal Cognitive Assessment (MoCA) score ≥ 26; and (3) right-handedness. The exclusion criteria were as follows: the presence of severe heart, liver, or kidney failure; malignant tumors; stroke; or other significant diseases. A total of 50 participants were recruited for this study (January 2023 to October 2023). In the data collection process, the fNIRS data of all participants underwent a rigorous quality check. The quality check criteria included: (1) Signal strength: Data is considered low quality if the signal cannot be recorded across all channels by the device; (2) Probe contact quality: Ensuring close contact between the fNIRS probes and the scalp; inadequate contact causing low signal and large motion artifacts in channels were excluded. During the quality check process, data from five participants were excluded because they did not have all effective channels recorded. Additionally, eight patients voluntarily withdrew from the experiment. Among them, one person withdrew due to a scheduling conflict, two people were concerned about potential radiation from fNIRS, and five people found it difficult to complete the experiment. The experimental procedure was approved by the Human Ethics Committee of Changzhou Dean Hospital, Changzhou, Jiangsu, China (CZDALL-2022-001) and registered on ClinicalTrials.gov (ChiCTR2200059252).

### 2.2 N-back paradigm

The n-back task was presented on a computer using E-Prime 3.0 (Psychology Software Tools Inc., PA, United States). The task included 1-, 2-, and 3-back conditions. The ratio of target to nontarget stimuli was 1:3, with a stimulus display time of 500 ms and a delay between the display times of 1,500 ms. A 20-s rest period was provided between blocks, and instructions were provided before each block. The participants pressed the “space” key on the keyboard to indicate a target stimulus. Each condition was repeated three times in a randomized order. Participants were required to practice before any formal measurements to ensure they fully understood the task. Each block consisted of 20 trials, with the reaction time and accuracy was recorded. We recorded both correct and incorrect responses for each stimulus in the accuracy.

### 2.3 fNIRS measurements

**Figure 1:**
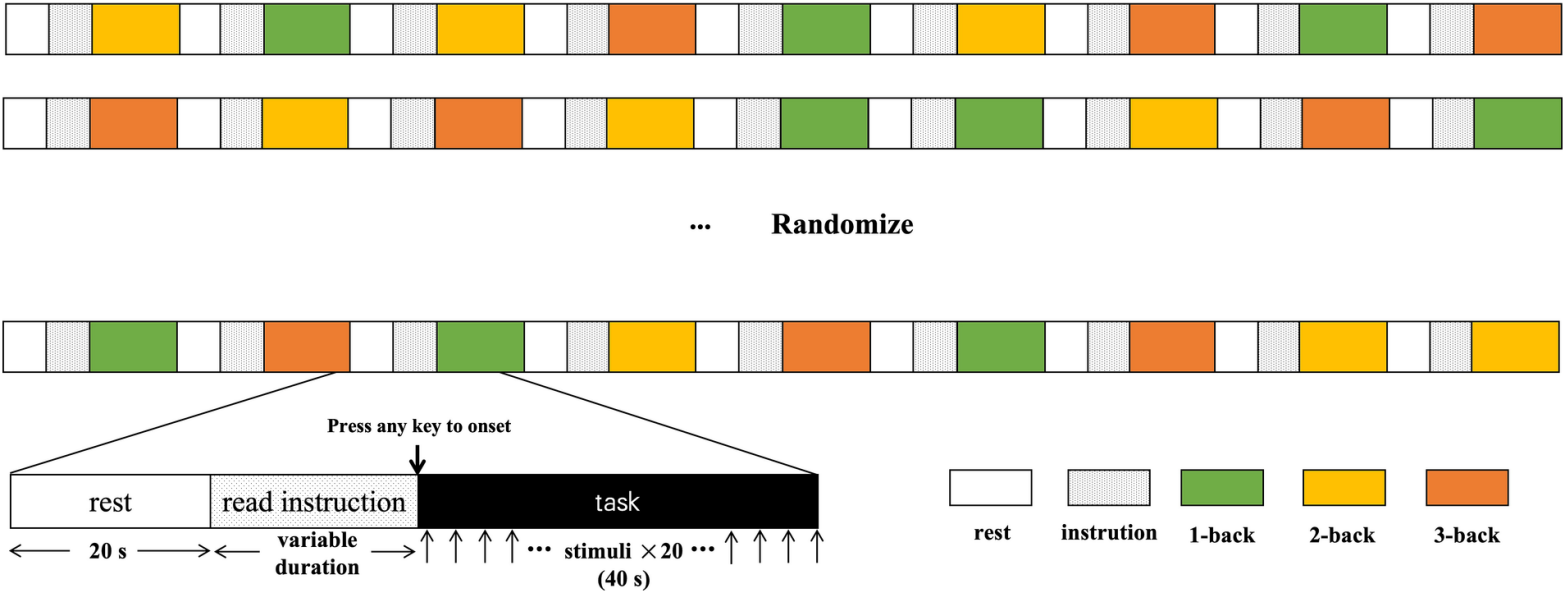
The diagram and process of n-back design.

Hemodynamic signals in the prefrontal lobes were recorded using the NirSmart fNIRS system (Danyang Huichuang Medical Equipment Co., Ltd., Zhenjiang, China) during n-back tasks. Near-infrared light (730 and 850 nm) was utilized at a sampling rate of 11 Hz. Forty-eight channels (15 source and 16 detector optodes) covered the cortex, with spatial information obtained from a brain template using an electromagnetic 3D digitizer (Patriot, Polhemus, Colchester, VT, United States) (Jurcak et al., 2007). Channels were mapped to the Montreal Neurological Institute (MNI) space according to the international EEG 10/20 coordinates (Jurcak et al., 2007) and classified based on Brodmann’s area coverage, with the highest percentage of channels representing functional areas. The inter-optode distance was 3 cm. Further details on channel placement and region of interest (ROI) are provided in Figure 1 and Table 1.

**Table 1:** Division of the ROIs for different functions.

Function	Brodmann area	Channel	ROI
Motor planning and execution	Right area 6	20	ROI1
	Left area 6	48	ROI2
Prefrontal associational integration	Right area 9/10/11/12	4 ~ 8/2 ~ 24/26 ~ 42	ROI3
	Left area 9/10/11/12	9 ~ 11/27/29/30/32/43 ~ 45/47	ROI4
Visual and auditory	Right area 21/22	1/2/17	ROI5
	Left area 21/22/42	13 ~ 16/33 ~ 35	ROI6
Limbic associational integration	Right area 38	3	ROI7
	Left area 38	12	ROI8
Taste processing	Right area 43	18	ROI9
	Left area 43	36	ROI10
Language production	Right area 44/45	19/31/46	ROI11
	Left area 37	37	ROI12

### 2.4 Procedure

Following the assessment, participants were asked to complete the three n-back tasks with increasing cognitive load while their brain activity was monitored using continuous-wave fNIRS. Prior to each task, participants received step-by-step instructions and practice trials with feedback. They were informed that their accuracy and reaction time would be recorded. The entire session lasted approximately 9 min, with rest breaks between tasks. The n-back procedure employs the following steps: First, there is a 20-s rest period. Next, participants read the instructions, and once they confirm their understanding, they press the spacebar to begin the testing phase. Each trial consists of 20 numbers displayed for 2 s each, during which participants can make judgments. The entire process lasts approximately 40 s, with 20-s rests between each trial. The 1-, 2-, and 3-back tasks are presented randomly three times each. The diagram is shown in Figure 1.

### 2.5 fNIRS data processing

**Figure 2:**
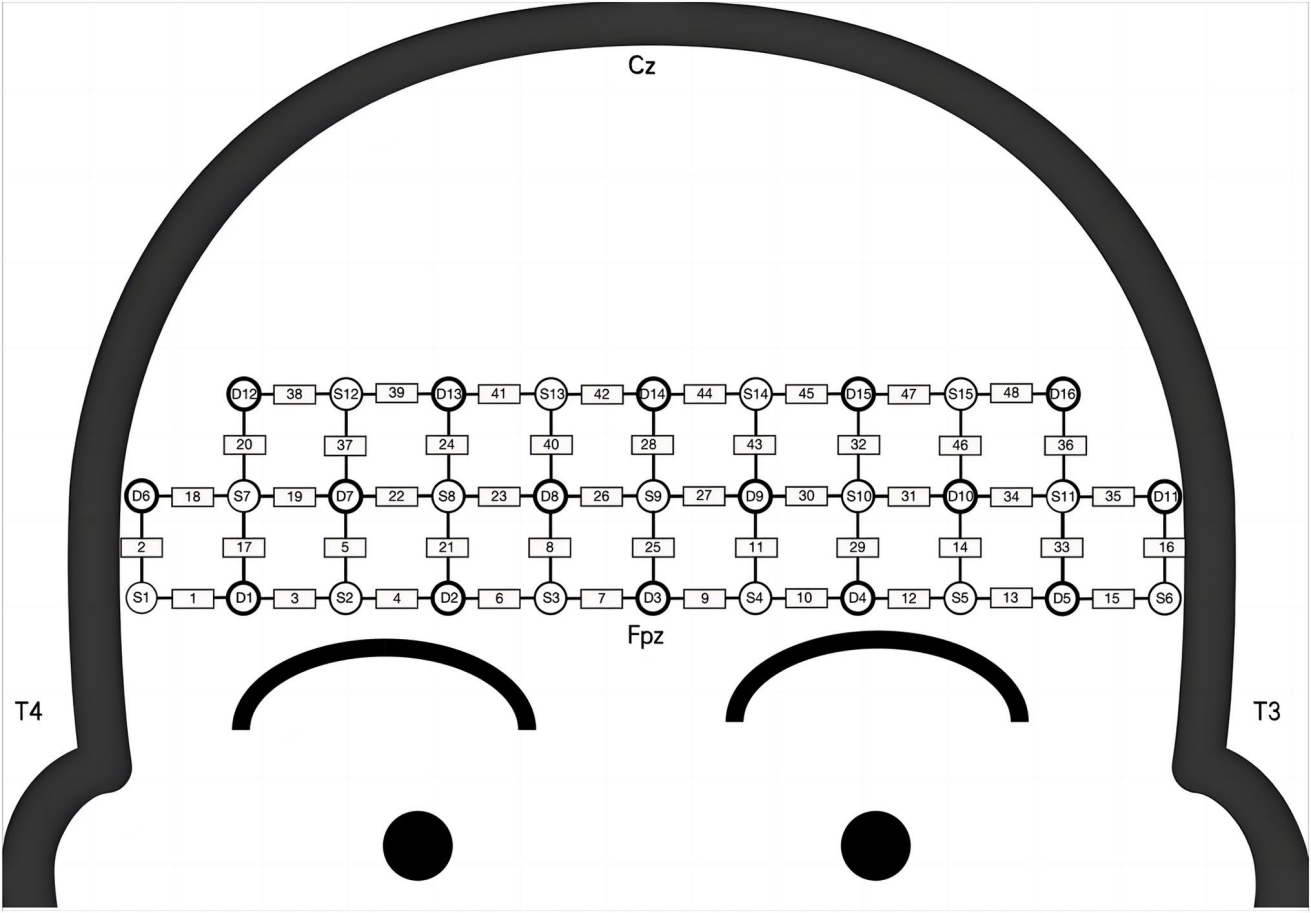
The attribution of sources, detections, and channels over the prefrontal lobe. S, source optode; D, detector optode; Cz, central zero; Fpz, frontal poles zero; T, temporal lobes.

fNIRS data were processed using NirSpark software (HuiChuang, Zhenjiang, China). Initially, raw signals were transformed into optical density, followed by motion artifact correction using a cubic spline interpolation algorithm (STDEV threshold = 6.0, AMP threshold = 0.5). A bandpass filter (0.01–0.1 Hz) was applied to attenuate low-frequency fluctuations and high-frequency noise while preserving task-specific frequency band of the neural signals (Hu et al., 2019). The filtered optical density was converted to blood oxygen concentration using the modified Beer–Lambert law (path length factor = 6) (Scholkmann and Wolf, 2013). Due to its superior signal-to-noise ratio compared with Deoxy-HB 30, we selected ΔHbO (the change of oxyhemoglobin) as the primary indicator (Strangman et al., 2002). A marker was set at 0 s when participants initiated each trial. We computed the average for the duration of 0–40 s as a block representing the hemodynamic response to the n-back task, with the last 2 s of rest serving as the baseline state. We then averaged the mean ΔHbO values from three repetitions to obtain an average response. These channel values within ROIs were further averaged to represent the activation of each brain region (Table 1 and Figure 2).

### 2.6 Statistical analysis

fNIRS data were analyzed using NirSpark software (HuiChuang, Zhenjiang, China). After obtaining the value of concentration changes, we used Matlab 2020a for further analysis. Initially, the Shapiro–Wilk test was conducted to assess conformity to normal distribution. For normally distributed data, we used a two-way ANOVA to determine significance, with *p*-values adjusted using false discovery rate (FDR) correction; hence, significance levels were established at 0.05. Then, we conducted a post-hoc analysis. The intergroup comparisons were made using independent t-tests, whereas intragroup comparisons were made using paired t-tests. Family-wise error (FWE) correction was applied with a significance level of 0.006, along with FDR correction due to the inclusion of multiple brain regions.

## 3 Results

Finally, 37 participants were included in the study. The general characteristics of the participants are presented in Table 2.

**Table 2:** Characteristics of participants.

	OG (*n* = 18)	YG (*n* = 19)
Gender (male/female)	9/9	7/12
Age (Mean ± SD)	64.28 ± 5.51	26.95 ± 3.12
Education years (Mean ± SD)	9.67 ± 2.20	15.47 ± 1.07
MoCA score (Mean ± SD)	28.33 ± 1.19	29.84 ± 0.37

ROI, region of interest; SD, standard deviation; OG, old group; YG, younger group; MoCA, montreal cognitive assessment.

### 3.1 N-back accuracy and reaction time

The accuracy was significantly influenced by both group (OG vs. YG; *F* = 17.12, *p* < 0.001) and task (1- vs. 2- vs. 3-back; *F* = 31.47, *p* < 0.001) factors, as well as their interactions effect (without statistical significance; *F* = 1.57, *p* = 0.21; Table 3). The reaction time was significantly influenced by group (OG vs. YG; *F* = 15.39, p < 0.001), but no statistical significance in task (1- vs. 2- vs. 3-back; *F* = 1.18, *p* = 0.31) and interactions effect (*F* = 0.11, *p* = 0.90; Table 4).

**Table 3:** Comparisons of accuracy performance under three conditions between the two groups.

N-back	Accuracy (mean ± SD)		Group		Task		Interactions	
	OG (*n* = 18)	YG (*n* = 19)	F	P	F	P	F	P
1-back	0.82 ± 0.10	0.91 ± 0.10						
2-back	0.73 ± 0.05	0.79 ± 0.08	17.12	<0.001*	31.47	<0.001*	1.57	0.21
3-back	0.72 ± 0.05	0.74 ± 0.05						

SD, standard deviation; OG, old group; YG, younger group; *p < 0.05.

**Table 4:** Comparisons of reaction time performance under three conditions between the two groups.

N-back	Reaction time *ms* (mean ± SD)		Group		Task		Interactions	
	OG (*n* = 18)	YG (*n* = 19)	F	P	F	P	F	P
1-back	469.34 ± 30.08	442.40 ± 48.74						
2-back	486.39 ± 39.26	453.05 ± 39.26	15.39	<0.001*	1.18	0.31	0.11	0.90
3-back	486.69 ± 41.24	450.65 ± 54.64						

SD, standard deviation; OG, old group; YG, younger group; *P < 0.05.

**Figure 3:**
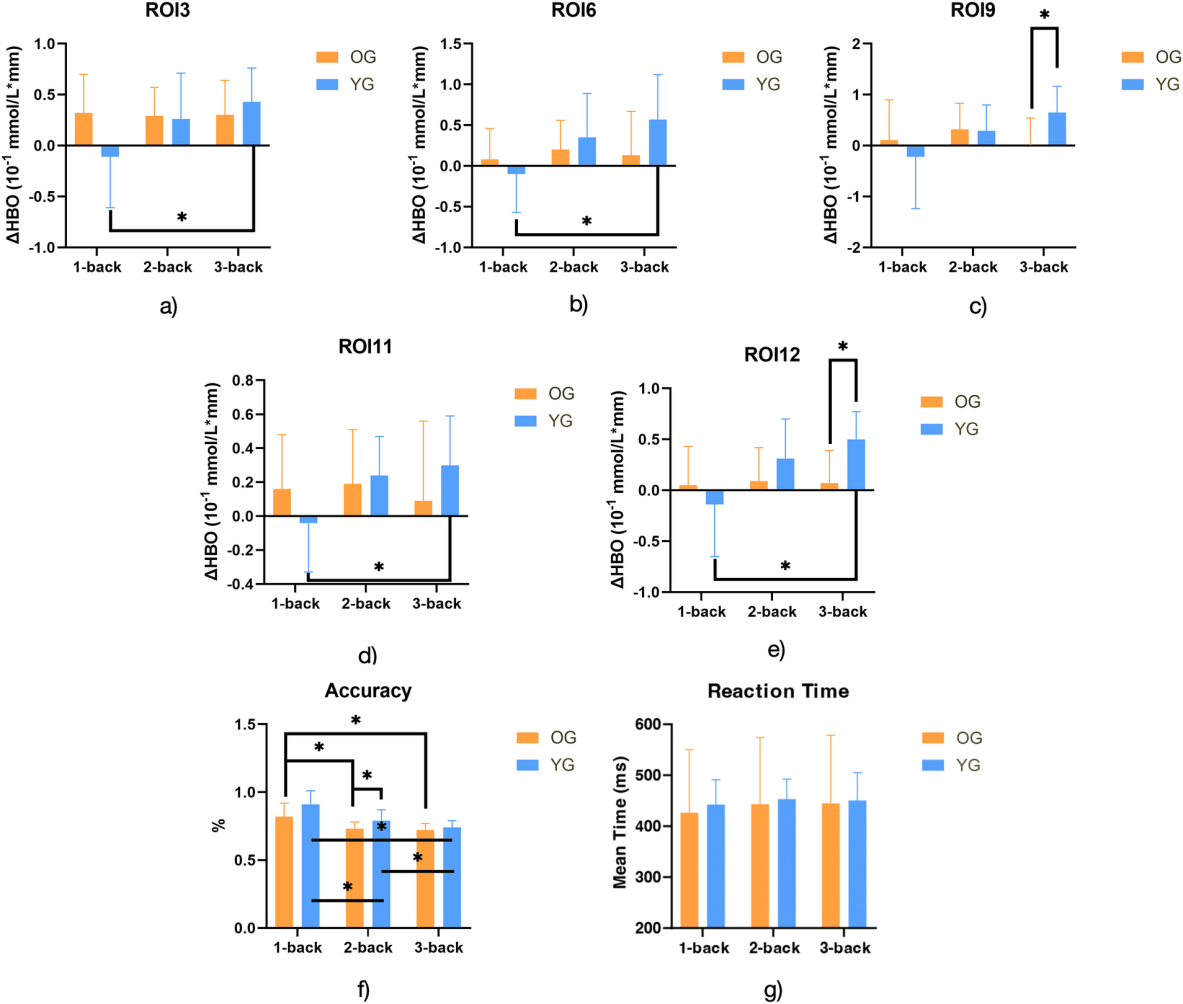
Bar charts for post-hoc analysis of ΔHbO in ROIs with significant differences, accuracy and reaction time. Notes: OG, older group; YG, younger, group; ΔHbO, the change of oxyhemoglobin; ROI3: Right prefrontal associational integration area; ROI6: Left visual and auditory area; ROI11: Right language production area; ROI12: Left language production area; *Adj-P/*p* < 0.006.

Post-hoc analysis of n-back accuracy (Figure 3) revealed significant differences between OG and YG across the tasks. When comparing 1-back vs. 2-back, both OG and YG exhibited significant differences in accuracy (*T* = 5.55, *p* < 0.001 for OG; *T* = 5.53, *p* < 0.001 for YG). For 1-back vs. 3-back, OG and YG also exhibited significant differences (*T* = 5.56, *p* < 0.001 for OG; *T* = 8.19, *p* < 0.001 for YG). Additionally, significant differences were found in the 2-back vs. 3-back condition for both OG and YG (*T* = 4.56, *p* = 0.000 for OG; *T* = −3.12, *p* = 0.004 for YG). No significant differences in post-hoc analysis of n-back reaction time (Figure 3).

### 3.2 fNIRS data

The analysis of ROIs revealed significant effects in certain regions and their interactions (Table 5). Specifically, ROIs 3, 4, 5, 6, 9, 11, and 12 demonstrated significant main effects as well as interactions, suggesting their substantial influence on the outcomes. The interactions were significant for the right prefrontal associational integration area (*F* = 5.17, Adj-*p* = 0.03), the left prefrontal associational integration area (*F* = 4.89, Adj-*p* = 0.03), the right visual and auditory area (*F* = 4.62, Adj-*p* = 0.03), the right taste processing area (*F* = 4.92, Adj-*p* = 0.03), and the right language production area (*F* = 3.83, Adj-*p* = 0.04). Moreover, the left visual and auditory area showed significant effects in both the main effect (*F* = 5.59, Adj-*p* = 0.03) and interactions (*F* = 3.91, Adj-*p* = 0.04). Similarly, the left language production area showed significant effects in both the main effect (*F* = 7.46, Adj-*p* = 0.01) and interactions (*F* = 6.41, Adj-*p* = 0.03). These results highlight the importance of these specific ROIs in influencing the observed outcomes and their interactions with other factors. However, no significant effects were found for the group or task factors.

**Table 5:** Comparisons of ΔHbO (10^−1^ mmol/L*mm) in 12 ROIs under three tasks between the two groups.

ROI	1-back (Mean ± SD)		2-back (Mean ± SD)		3-back (Mean ± SD)		Group		Task		Interactions	
	OG	YG	OG	YG	OG	YG	F	Adj-P	F	Adj-P	F	Adj-P
1	−0.04 ± 0.59	−0.21 ± 0.86	0.17 ± 0.47	0.17 ± 0.30	−0.02 ± 0.59	0.36 ± 0.52	0.39	0.71	3.11	0.11	2.14	0.13
2	0.12 ± 0.32	0.04 ± 0.66	0.13 ± 0.50	0.42 ± 0.47	0.08 ± 0.45	0.34 ± 0.42	2.83	0.40	1.59	0.25	1.76	0.18
3	0.32 ± 0.38	−0.11 ± 0.50	0.29 ± 0.28	0.26 ± 0.45	0.30 ± 0.34	0.43 ± 0.33	2.26	0.40	4.27	0.07	5.17	0.03*
4	0.28 ± 0.39	−0.12 ± 0.45	0.10 ± 0.37	0.20 ± 0.44	0.18 ± 0.38	0.29 ± 0.34	0.71	0.69	1.44	0.26	4.89	0.03*
5	2.43 ± 3.07	−0.63 ± 5.18	1.76 ± 2.89	2.94 ± 3.94	1.89 ± 3.26	3.56 ± 3.14	0.01	0.92	2.53	0.13	4.62	0.03*
6	0.08 ± 0.38	−0.10 ± 0.47	0.20 ± 0.36	0.35 ± 0.54	0.13 ± 0.54	0.57 ± 0.55	2.25	0.40	5.59	0.03*	3.91	0.04*
7	0.20 ± 0.55	−0.09 ± 0.66	0.21 ± 0.65	0.36 ± 0.57	0.21 ± 0.74	0.51 ± 0.53	0.18	0.81	2.41	0.13	2.30	0.13
8	0.23 ± 0.59	0.01 ± 0.53	0.37 ± 0.74	0.56 ± 0.83	0.20 ± 0.90	0.83 ± 0.87	1.94	0.40	3.00	0.11	2.90	0.09
9	0.11 ± 0.79	−0.22 ± 1.02	0.32 ± 0.51	0.29 ± 0.51	0.01 ± 0.53	0.65 ± 0.51	0.53	0.70	3.75	0.08	4.92	0.03*
10	0.19 ± 0.35	0.04 ± 0.72	0.16 ± 0.37	0.28 ± 0.71	0.12 ± 0.38	0.51 ± 0.49	1.39	0.48	1.31	0.27	2.39	0.13
11	0.16 ± 0.32	−0.04 ± 0.29	0.19 ± 0.32	0.24 ± 0.23	0.09 ± 0.47	0.30 ± 0.29	0.08	0.84	2.62	0.13	3.83	0.04*
12	0.05 ± 0.38	−0.14 ± 0.51	0.09 ± 0.33	0.31 ± 0.39	0.07 ± 0.32	0.50 ± 0.27	4.59	0.40	7.46	0.01*	6.41	0.03*

ROI, region of interest; SD, standard deviation; OG, old group; YG, younger group; ROI1: Right motor planning and execution area; ROI2: left motor planning and execution area; ROI3: right prefrontal associational integration area; ROI4: left prefrontal associational integration area; ROI5: Right visual and auditory area; ROI6: left visual and auditory area; ROI7: Right limbic associational integration area; ROI8: left limbic associational integration area; ROI9: Right taste processing area; ROI10: left taste processing area; ROI11: Right language production area; ROI12: left language production area; *Adj-*P* < 0.05.

Post-hoc analysis on n-back accuracy (Figure 3) showed significant increases in brain activation within YG only in the 1- vs. 3-back comparison, specifically in the right prefrontal associational integration area (*T* = −3.99, *p* = 0.002), the left limbic associational integration area (*T* = −4.54, *p* = 0.002), the right language production area (*T* = −4.04, *p* = 0.003), and the left language production area (*T* = −4.72, *p* = 0.002). Intergroup comparisons revealed significant differences between OG and YG only in the 3-back condition, where the right taste processing area (*T* = −3.74, *p* = 0.004) and the left language production area (*T* = −4.37, *p* = 0.001) exhibited lower activation in OG.

## 4 Discussion

Our results revealed that young adults exhibited a gradual decline in accuracy when performing n-back tasks of varying difficulty, whereas older adults performed better on the 1-back task than the 2- and 3-back tasks, with no significant difference observed between the latter two tasks. Furthermore, intergroup comparisons showed no significant difference between the groups on the 1- and 3-back tasks, whereas the 2-back task exhibited significantly lower performance in OG than in YG. These findings suggest that older adults may reach their cognitive limit between the 1- and 2-back tasks. In terms of changes in ΔHbO values, we observed an increase in activation across the entire PFC, including parts of the temporal lobe, in YG when comparing between the 1- and 3-back tasks. Conversely, among older adults, cognitive-related brain regions showed a trend of decreased activation with decrease in task difficulty. Intergroup comparisons under different conditions revealed a significant decrease in activation in the right speech and left taste areas among older adults.

In the n-back task, we observed a decline in accuracy among both groups with increasing task difficulty, aligning with prior research findings (Mattay et al., 2006; Vermeij et al., 2012; Yeung and Han, 2023). This decline correlates closely with the inherent difficulty of the n-back task itself. In a standard 1-back task, various processes such as identification, maintenance, and updating come into play, as each stimulus sets the criterion for the next trial. Thus, the stimulus needs to be retained, and the criterion adjusted for each subsequent trial. Conversely, a typical 2-back task involves not only identification, maintenance, and updating but also the inhibition of distractors. This is because, between each criterion and potential target, an extra stimulus appears, which must be retained as well as suppressed if matched on the next trial (Yaple et al., 2019). The 3-back task involves a more complicated process. Interestingly, we observed a divergence in performance trends between the two age groups: while performance in elderly individuals plateaued at the 2-back task, that in young participants plateaued at the 3-back task, during which they exhibited similar performance as the elderly individuals. This observation is closely tied to our metric calculation approach. Notably, our assessment included not only responses to stimuli but also accuracy calculations for nontarget stimuli conditions. Consequently, even in the absence of a response, a baseline accuracy of 0.75 remained. This phenomenon was particularly evident in the 3-back condition, where both elderly and young participants achieved similar accuracy of approximately 0.75, suggesting a potential performance limit at this level.

The findings in OG suggest that the 2-back task may have pushed older adults’ cognitive load to the limits of their cognitive capacity. This observation is corroborated by neural-level findings as well. Despite notable differences in brain regions across different conditions, bilateral PFC areas associated with working memory exhibited the highest activity levels in the 1-back condition, whereas activity levels in the 2- and 3-back conditions were lower than those in the 1-back condition. Prior research has also indicated that as task cognitive demands rise, the utilization of neural resources to meet these demands increases, ultimately enhancing cognitive performance (i.e., compensation). However, this heightened recruitment cannot be sustained under high loads, as the neural resource capacity reaches its limit, resulting in a decline in cognitive performance (Mattay et al., 2006; Nyberg et al., 2009).

For reaction time performance, we found significant differences between the groups, but no significant differences across different n-back difficulty levels. However, post-hoc analysis did not show any significant differences. First, post-hoc analysis took stringent FWE corrections, which excluded some potentially significant differences. Additionally, a previous study (Verhaeghen and Basak, 2005) has shown that reaction time is less sensitive than accuracy, which is consistent with our findings. Considering that each stimulus in our experiment was presented for only 2 s, participants had limited time for judgment and reflection, leading to prioritizing timely decisions. Therefore, accuracy seems to be a more suitable metric for the exploration of differences between OG and YG in our n-back task.

Particularly among older adults, these phenomena have been observed consistently across age cohorts. Older adults tend to engage in over-recruitment as a compensatory mechanism at lower cognitive loads. However, as cognitive demands increase, they eventually reach the limits of their neural resources, resulting in diminished performance (Reuter-Lorenz et al., 2000). When the cognitive load reaches a certain level, the “compensation” strategy is no longer sufficient to explain this situation, and older adults often begin to adopt the so-called “abandonment” strategy. Therefore, their brain activation levels decrease under more challenging tasks, whereas their performance shows little difference. A previous study (Reuter-Lorenz and Cappell, 2008) reported compensatory activation in the right PFC among older adults during lower demand tasks. However, this activation diminished as task difficulty increased, leading to underactivation at higher task demands. In the present study, OY reported a gradual decline in performance, accompanied by the continuous increase in prefrontal activation, which aligns with previous findings (Ayaz et al., 2012; Csipo et al., 2021). We also noticed that differences were seen only between the 1- and 3-back conditions. One possible explanation for this can be drawn from Nyberg et al.’s study (Nyberg et al., 2009), which found that activation peaked during the 3-back task in healthy young participants with high working memory capacity, whereas among those with low working memory capacity, it peaked during the 2-back task. Since our study did not differentiate participants based on their cognitive abilities, we were unable to detect differences in the progression of task difficulty.

In comparison between the two groups, we observed an interesting phenomenon. While the PFC is often closely associated with working memory and other cognitive tasks and is relatively sensitive (Yaple et al., 2019), our results did not show significant differences in bilateral PFC activation between the groups. This may be attributed to the large number of comparisons and observed brain regions in our study, as well as the strict corrections applied. A simpler experimental design, as seen in previous studies, might have yielded similar findings. However, even with stringent corrections, we found significant differences in some areas unrelated to cognition, particularly under the 3-back task condition. Moreover, we observed a significant decrease in the activation of brain regions related to taste and language in older adults, as shown in a previous fMRI study (Rajah and McIntosh, 2008). During the execution of high-cognitive-load tasks, older adults often exhibit functional compensation by recruiting increased activation in the prefrontal and precuneus regions. However, this compensation is accompanied by a significant decrease in activation in partially unrelated temporal and parietal regions, suggesting deficits in activating reserve resources. Similar phenomena have also been observed in patients with cancer-related cognitive dysfunction (Root et al., 2021). We covered partially unrelated areas in this study because we utilized additional channels. However, this arrangement unexpectedly led us to discover regions that may be more sensitive than the bilateral PFC. As older adults exhibit similar activation to young adults in simple tasks, and bilateral PFC activation increases cease to be significant once the cognitive load limit is reached, the significant decrease in activation of unrelated brain areas may likely serve as a sensitive observation target. Studies on the neural effects of the n-back task have often reported differences in activation of regions such as the DLPFC, but reports on unrelated brain regions are rare, which is because unrelated brain regions are typically excluded during ROI selection. Additionally, there may be some reporting bias in describing results. One possible explanation is that the mechanism of fNIRS measurements is still based on neurovascular coupling theory (Yeung and Chu, 2022). Cognitive abilities in older adults are severely limited, and when activation (blood flow) in cognitive brain regions reaches its limit, maintaining a high level of activation in these areas may only be possible by reducing blood flow in the surrounding unrelated brain regions. This could be indicative of a decline in brain blood flow reserve capacity. Our finding could potentially facilitate the identification of auxiliary diagnostic indicators for cognitive decline in older adults in the future.

### 4.1 Limitations

First, our sample size was relatively small, which is a common challenge faced by most studies. Preliminary screening with small samples is aimed at providing potential evidence for the success of future large-sample experiments. Second, while fNIRS is convenient to use, not all individuals’ signals can be reliably identified, with some participants experiencing low signal quality during data collection. At the same time, the spatial resolution of fNIRS is lower than that of fMRI. While fMRI offers uniform resolutions of 1–3 mm, the spatial resolution of fNIRS is constrained by the number of detector optodes and the light scattering between the emitter and detector. Current systems use detectors spaced approximately 3 cm apart from the illuminating optode. Compared to EEG, the temporal resolution of fNIRS is also relatively lower. The sampling rate of fNIRS is typically around 10 Hz, while EEG can reach several hundred to even thousands of Hz. Future experiments should be designed to leverage the strengths of near-infrared spectroscopy while avoiding its limitations in spatial and temporal resolution. Third, although the difficulty levels in n-back tasks were clearly distinguishable, there are limitations to the evaluation metrics of n-back tests. Regardless of whether calculations include responses to nontarget stimuli, there is a certain performance plateau effect, indicating the need to explore better tasks in the future. Forth, included responses to both stimuli and nontarget stimuli into accuracy calculations could bring a floor effect. This approach reduces the differences between the two groups. However, focusing only on stimuli often results in missing values, which severely be a threat to the statistical method. Future experiments should consider whether better metrics and statistical methods should be used. Finally, the participants we included needed to be able to understand and complete the n-back task, which requires a certain level of cognitive ability. As a result, a significant portion of elderly individuals was excluded. This limitation affects the generalizability of our findings. Therefore, for elderly populations with cognitive impairments, a more suitable assessment may be necessary in the future studies.

## 5 Conclusion

In summary, our study revealed differences in cortical activation between elderly and young individuals during n-back tasks, especially under the 3-back condition. Additionally, the DLPFC, which we have traditionally focused on, may not be sensitive to neuroimaging tools such as near-infrared spectroscopy, and activation in surrounding irrelevant brain regions could serve as more effective observation targets. Future large-sample experiments may provide new evidence for screening age-related cognitive decline.

## Data Availability

The raw data supporting the conclusions of this article will be made available by the authors, without undue reservation.
